# Novel archaeological and palaeontological findings in cave and palaeoriver landscapes of inland northeast Arabia

**DOI:** 10.1371/journal.pone.0337005

**Published:** 2025-11-20

**Authors:** Huw S. Groucutt, Mathew Stewart, Faisal Al-Jibreen, Mesfer Al-Qahtani, Mahmoud Al-Shanti, Eric Andrieux, James Blinkhorn, Nicole Boivin, Paul S. Breeze, Nick Drake, Abdullah Memesh, Yahya Mufarreh, Gilbert Price, Eleanor M. L. Scerri, Nils Vanwezer, Hubert Vonhof, Iyad Zalmout, Abdullah M. Alsharekh, Michael D. Petraglia

**Affiliations:** 1 Department of Classics and Archaeology, University of Malta, Msida, Malta; 2 Institute of Prehistoric Archaeology, University of Cologne, Cologne, Germany; 3 Australian Research Centre for Human Evolution, Griffith University, Brisbane, Australia; 4 Heritage commission, Ministry of Culture, Riyadh, Saudi Arabia; 5 Department of Anthropology, University of Pittsburgh, Pittsburgh, Pennsylvania, United States of America; 6 Geotourism Department, Saudi Geological Survey, Jeddah, Saudi Arabia; 7 Department of Archaeology, Durham University, Durham, United Kingdom; 8 Human Origins Research Group, Department of Archaeology, Classics, and Egyptology, University of Liverpool, Liverpool, United Kingdom; 9 Human Palaeosystems Group, Max Planck Institute of Geoanthropology, Jena, Germany; 10 Department of Archaeology, Max Planck Institute of Geoanthropology, Jena, Germany; 11 School of Social Science, The University of Queensland, Brisbane, Australia; 12 School of Environment and Science, Griffith University, Brisbane, Australia; 13 Department of Geography, King’s College London, London, United Kingdom; 14 Survey and Exploration Centre, Geological Programs, Saudi Geological Survey, Jeddah, Saudi Arabia; 15 School of The Environment, The University of Queensland, Brisbane, Australia; 16 State Office for Heritage Management and Archaeology, Saxony-Anhalt, Germany; 17 Department of Climate Geochemistry, Max Planck Institute for Chemistry, Mainz, Germany; 18 Department of Archaeology, College of Tourism and Archaeology, King Saud University, Riyadh, Saudi Arabia; 19 Human Origins Program, National Museum of Natural History, Smithsonian Institution, Washington, DC, United States of America.; Universita degli Studi di Ferrara, ITALY

## Abstract

Knowledge about environmental change and the evolutionary history of hominins in Arabia has been rapidly developing over the last two decades. Interdisciplinary research on humans and environments across the vast and heterogenous landmass of the Arabian Peninsula remains, however, highly spatially uneven. Here we present the results of archaeological, hydro-geological, and palaeontological research in inland northeastern Arabia, a poorly studied area with diverse landscape features including caves, palaeorivers, and chert outcrops. Hominin use of the landscape appears to be sparse in comparison to other regions of Arabia, though archaeological evidence spanning from the Lower Palaeolithic to the historic era was identified, including finds from the Middle Palaeolithic, which is the most well represented period. The caves of inland northeast Arabia contain a rich record of past climate change in the form of speleothems, as well as abundant faunal assemblages. Our survey results highlight the significant potential of these records to cast light on environmental, faunal, and cultural changes over time while demonstrating regional variation across Arabia.

## Introduction

While it is increasingly recognised that the Arabian Peninsula has a rich archaeological record [1,2], it is only in the last two decades that significant advances have been made in understanding the evolutionary history of hominins in the region. The application of chronometric dating methods has been crucial, providing a temporal framework for climate change, human demography, and behavioural variability in the region [e.g., 3–10]. Likewise, considerable advances have been made in elucidating Quaternary palaeoenvironmental change [e.g., 11–15]. These findings have cast light on hominin demographic and behavioural changes from the Middle Pleistocene to the Holocene [e.g., 6–8,16–20]. Furthermore, several studies have evaluated the relationship between past human societies and the diverse and changing landscapes, ecology, and hydrology of Arabia through time [e.g., 12,13,21–26]. While recent studies have improved knowledge on the environmental and hominin history of Arabia, significant research biases and lacunae remain in understanding this vast and varied region. Most archaeological research has focussed on the southeast and northwest of Arabia, with vast swathes of the peninsula still virtually unstudied. One such area is inland northeast Arabia, defined here as the area east of the Ad Dahnā desert, between Qatar and Kuwait, covering some 50,000 km^2^. To our knowledge, no Pleistocene archaeological sites have been reported from this region. Likewise, little is known from the Holocene, aside from Ubaid sites along the Gulf coast [e.g., 27,28,29].

With respect to the character and landscape position of Arabian archaeological sites, most are open air sites, with limited information about human occupation of caves and rockshelters. Whether this reflects behavioural choices in occupations, or simply that archaeological investigations in Arabia remain undeveloped, is currently unclear. Particularly in Saudi Arabia, the largest country in the region, recent archaeological research has focussed on open air settings such as palaeolakes [e.g., 7], as these have emerged as a rich source for palaeoenvironmental, palaeontological, and archaeological information.

One theme that has emerged from recent studies in Arabia is that a deeper understanding of the relationship between climate, environment, and human occupation history is needed. At a broad scale, ‘Green Arabia’ phases reflect increased rainfall relative to evaporation, and these were the periods in which human populations—as well as those of some animals—were able to spread into and through this generally arid region [e.g., 7,30]. Within this broad framework, though, more specific elements of the palaeohydrology of Arabia are in need of examination. Speleothems, for example, offer an important direct record of local precipitation [e.g., 13,15,31]. However, the relationship between regional climatic changes and variation in water availability for humans is not necessarily simple. For instance, as highlighted by Groucutt [32], volcanic activity and corresponding landscape changes can lead to variation in local hydrology, independent of changes in precipitation and evaporation. Likewise, studies of palaeorivers indicate that fluvial networks were a crucial aspect of the Arabian palaeoenvironment, not just local rainfall [e.g., 21,22]. Aquifer dynamics are yet another key element in understanding how past climate change would have impacted human societies. Precipitation on impermeable bedrock in steep terrain will have very different impacts compared to rainfall on flat, permeable geology, where large aquifers can form. In the case of sand seas such as the Nefud Desert, the high infiltration rates of sand mean that in humid periods groundwater is an important resource and thus aquifer dynamics play an important role in linking precipitation to water accessibility. Despite this importance, our understanding of aquifer dynamics in relation to human prehistory in Arabia remains limited. Furthermore, the interplay between changing precipitation and evaporation patterns and water availability is complicated by the presence of large catchments and aquifer systems.

Alongside, and often bridging, records of past human populations and changing climate, recent studies have begun to develop an understanding of the palaeontological record of Arabia. The region’s faunal diversity offers insights on the shifting characteristics of the landscape through time, Arabia’s biogeographical connections with adjacent areas, as well as processes of domestication which have transformed human-animal relationships in the recent past.

Pleistocene faunal remains, including taxa such as *Palaeoloxodon* (straight tusked elephants) and hippopotamus [e.g., 6,7,9,30,33,34], serve as key indicators of past ‘Green Arabia’ periods. Holocene faunal remains, while informative, are often poorly preserved [e.g., 35,36,37,38]. However, certain ritual stone structures that contain human-deposited animal remains in sheltered conditions offer much better preservation [39,40,41], though such assemblages have a strong anthropogenic filter. Beyond inland and ritual contexts, sites near the Gulf coast often contain abundant faunal remains with a strong marine element [42]. While the rapidly expanding palaeontological and zooarchaeological records of Arabia offers important insights, significant biases remain, particularly concerning preservation and anthropogenic filters. Recent work in the Umm Jirsan lava tube in northwest Arabia shows how such underground settings offer exceptional environments for bone preservation in the context of carnivore (particularly hyena) dens [9,43], in contrast to open arid settings where the bone preservation is otherwise extremely poor. In addition, species depicted in rock art provide another way of reconstructing past ecosystems [e.g., 35]. Despite the inherent biases in these various records, by ‘triangulating’ the various lines of evidence we can develop a more comprehensive understanding of Arabia’s changing ecosystems over time and their broader implications.

## Study area and aims

**Fig 1:**
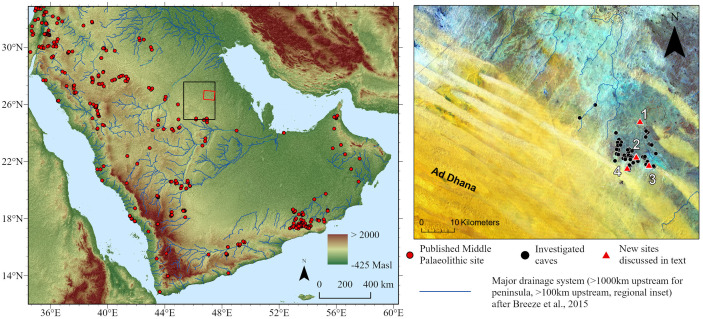
Map of Arabia showing study area and key new sites. Left, overview of the topography of the Arabian Peninsula (STRM 90 m data [44]), with Middle Palaeolithic sites of Arabia and the Levant (updated from [2]) displayed. The location of the inset figure is displayed as the red bounding box, and that of Fig 3 as the black bounding box. Right: Inset displaying a Landsat TM FCC median composite for the study area, used to highlight different geomorphological features (dunes in yellow, carbonate bedrock in grey/cyan) with caves (black points) and key sites discussed in the text (red triangles) overlain. Palaeodrainage data derived from HydroSHEDS [45], following [21] is overlain. Sites discussed in the text are labelled as follows: 1 = EP19.90; 2 = 19.86 & 19.1; 3 = 19.8 & 19.91; 4 = Murrubah.

Considering this, northeast Arabia (Fig 1) emerges as an important area of study for developing more balanced records which can allow the evaluation of different models for prehistory in the Arabian Peninsula. Northeast Arabia is currently a ‘blank spot on the map’ in terms of modern archaeological field research. Yet, it is an area with a multitude of caves and palaeorivers, offering an opportunity to examine changing landscapes, hydrological dynamics, and human occupations.

**Fig 2:**
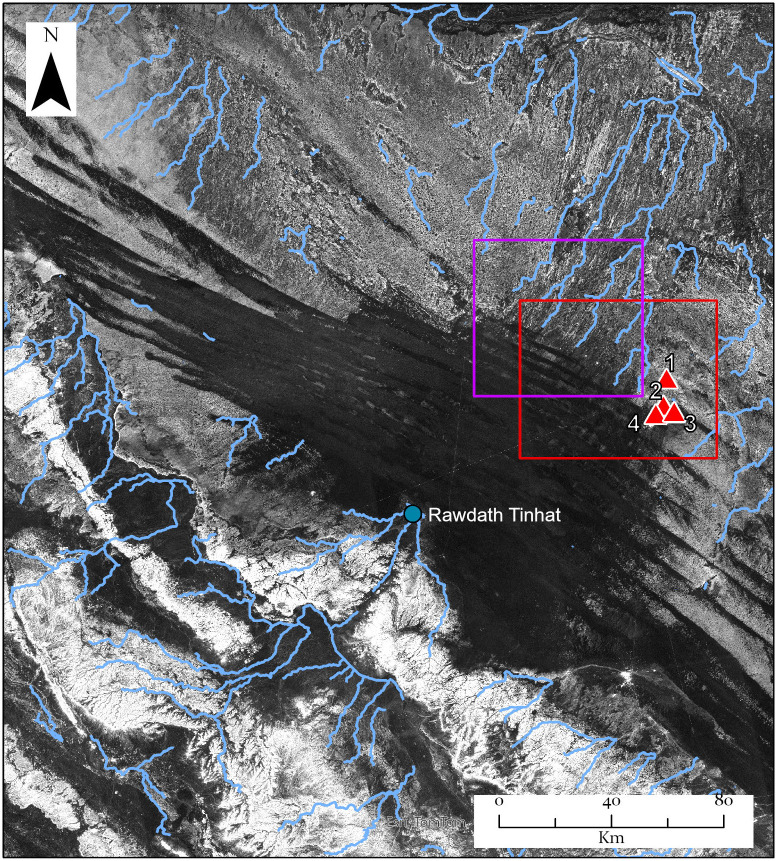
Palaeohydrology of the study area. From HH polarisation ALOS PALSAR radar data [49] which reveals palaeochannels buried beneath shallow sand, and with channels with a preserved topographic expression overlain (calculated from the HydroSheds dataset for channels with >100 km upstream accumulation area). Sites discussed in the text are overlain and labelled as in Fig 1. The red rectangle marks the location of the inset in Fig 1, while the purple extent shows the limits of the detailed zoom in on this data presented in Fig 3. The JAXA Global PALSAR-2/PALSAR/JERS-1 Mosaic and Forest/Non-Forest maps data used for this paper have been provided by the Japan Aerospace Exploration Agency (https://www.eorc.jaxa.jp/ALOS/en/palsar_fnf/data/index.htm).

**Fig 3:**
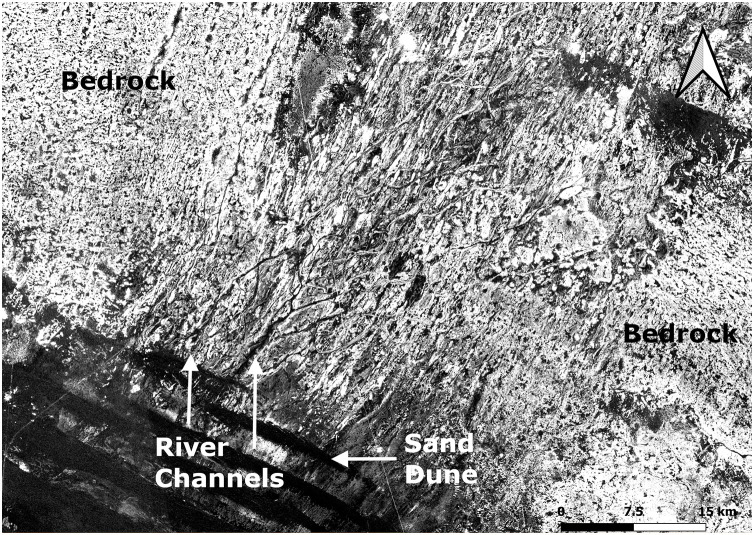
PALSAR HH radar image of the study region. The river channels are seen emerging from under the dunes of the Ad-Dahnā. Sand dunes are the dark features in the bottom left of the image whilst the black and white lines are palaeoriver channels, the black ones being channels cut into the limestone bedrock and the light ones the inverted river channels.

Here we report on interdisciplinary research in the As Sulb plateau (Figs 1–3), in northeastern/north-central Saudi Arabia, forming part of the larger As Summan Plateau. The geology of the study area mainly consists of the Palaeocene-Early Eocene Umm er Radhuma Formation and an overlying, unnamed, Miocene-Pliocene unit [46–48]. The former consists of shallow marine limestone and dolomite and is relatively chert-rich in and gypsum-rich in parts. The overlying Miocene-Pliocene formation consists of a heterogenous series of calcareous sandstone, sandy marl, shale, and lacustrine limestone. This unit is wedge-shaped, being thickest in the south (~300 m) where it passes under the sands of the Empty Quarter, tapering off to the west and to the east where it grades into marine facies.

The As Sulb area is highly karstic, and previous studies have touched upon the large number of caves in the area [50,51]. In some cases, such as UPM Cave [48] and Dahl Sultan [50], the caves contain hundreds of metres of passages. A pilot project identified the rich record of speleothems in the area [31], followed by a major new study by our team, reporting an eight million year long climate record [15].

Preliminary work on bone assemblages from As Sulb caves [52] has been conducted. Most of the fauna reported by Bauer [52] consists of micromammals, such as gerbils, but also included larger fauna such as gazelles, camels, and foxes. The discovery of pygmy gerbil (*Gerbillus henleyi*) extended the known historic distribution of this species some 830 km to the northeast, while the presence of the Euphrates jerboa (*Allactaga euphratica*) extended its known historic distribution some 250 km southwards. At Murubbeh Cave (B7), large numbers of animal bones were found, with some interpreted as reflecting striped hyena (*Hyaena hyaena*) accumulations based on the tooth marks and the extent of damage to the bones. Among the finds was a naturally mummified fox, radiocarbon dated to ca. 150 AD (calibrated) [52].

The As Sulb area is of interest given implications for palaeohydrology and aquifer dynamics. While this area of Saudi Arabia is currently arid, with less than 100 mm annual rainfall on average, hydrological research indicates that around 47% of precipitation enters aquifers through the cave system given the highly karstic and faulted nature of the area [53–55]. The Umm er Radhuma aquifer is one of the most important in Saudi Arabia. It extends from the inland karstic areas of northeast Saudi Arabia, including the current study area, to the Gulf Coast where large aquifer-fed springs occur. It was previously unclear if this water reflected aquifer accumulation during previous humid periods, as suggested groundwater ages of between 26–20,000 years ago have been reported [56], or if it is also being recharged during the current period. Hoetzl [54] found that karstification aided water retention, and occasional storm events could lead to significant aquifer recharge in this generally arid area. In fact, extrapolating the directly studied area to the total catchment area, produces an estimate of recharge which is similar to the outflow from the Gulf Springs, some 200–250 km east [54]. A more recent study likewise supported large flows of water into the caves in the As Sulb plateau [55]. On a more local level, caves have provided water for people in the As Sulb area [56,57]. This is indicated by rope grooves on the edges of the some of the deep shafts suggesting their use as wells, such as at Dahl as Hashami [56].

## Materials and methods

Our interdisciplinary team conducted field research in northeast Arabia in 2019. The focus was on the As Sulb area, near the village of Shawyah, which is around 200 km inland, and roughly halfway between Qatar and Kuwait. Prior to fieldwork, remote sensing and GIS analysis was used to investigate the landscape and paleohydrology of the study area to divide it into areas with different palaeohydrological and landscape characteristics so their archaeological and palaeontological potential could be investigated. A map of the caves was provided by the Saudi Geological Survey and the region surrounding them selected for survey (Fig 1). The cave distribution was compared to the geology of the area [47,48] to investigate its potential control on the presence of caves. Palaeolake and river deposits were mapped using the methods described in Breeze and colleagues [22]. As this mapping only shows channels with a preserved negative topographic expression, we interpreted PALSAR (Phased Array L-band Synthetic Aperture Radar) imagery to locate and investigate relict river courses that are now left stranded in the landscape. A sample of these rivers and lakes were then surveyed in the field. In other parts of Arabia, this method has proven effective for targeting archaeological sites, as these areas offered ready access to fresh water and other essential resources during past periods of higher humidity.

For the limestone plateau where the caves are located, we conducted vehicle-based and pedestrian surveys. We targeted as many caves as possible, including those sites identified through satellite imagery, and those discovered during reconnaissance, ultimately visiting 79 in total. Newly identified localities were given a unique code, beginning with EP (Eastern Province, e.g., EP19.1). Where possible, we explored these caves to evaluate their scientific potential. In most cases the caves have a similar structure, with a vertical entrance shaft and variable lengths of accessible horizontal passages, often parallel to the geological bedding plane and sometimes extending hundreds of meters. In some cases, it was impossible for our team to enter the caves, and future work in the area should make use of caving methods such as single rope techniques (SRT) to access some of the more challenging caves.

Many of the caves we visited were found to contain abundant animal bones, including some very recent introductions. In three cases we identified significant palaeontological assemblages – at Murubbeh Cave, and two sites which as far as we are aware have not been previously scientifically reported, EP19.8, and EP19.91. We collected small samples from these localities for taxonomic identification and radiocarbon dating. From Murubbeh, selected diagnostic faunal remains were targeted for collection from the northwest and northeast passages, while at EP19.8 and EP19.91 bones were retrieved from three discrete caches of bones at each site. Taxonomic identifications were facilitated by relevant literature [58] and osteological collections housed at the Royal Museum for Central Africa, Belgium. As the collection of animal remains was not systematic, and no excavation or sieving was undertaken, the assemblages are somewhat biased, particularly with respect to small species and bone fragments. 3D models were created using photogrammetry, and examples can be seen at https://sketchfab.com/UQ_SEES/collections/fossils-from-the-as-sulb-plateau-saudi-arabia-090b8fcfb54845939d624ad97c500ff9.

Radiocarbon dating of bone samples was subsequently carried out at the Scottish Universities Environmental Research Centre (SUERC) Radiocarbon Dating Laboratory. Collagen was extracted using a modified Longin method and the dried extract subjected to combustion, reduction, and AMS measurement following established methods [59].

Surveys were also conducted in the general landscape, such as areas near caves and chert sources. In addition, we targeted a large stone structure (EP19.90) which had been identified via satellite imagery, as well as palaeorivers just to the northwest. In most cases lithics (knapped stone tools), which form the main category of prehistoric archaeological evidence, were found at a very low density on the surface. Accordingly, representative samples of lithics were collected at particular localities. In one case, we found a denser concentration of lithic artefacts (EP19.1), which we systematically collected.

## Ethics statement

All necessary permits were obtained for the described study, which complied with all relevant regulations. Collection and analysis of all specimens were done under a permit granted by the Saudi Heritage Commission. All samples will be held for long term storage with the Heritage Commission, Riyadh, Saudi Arabia.

Additional information regarding the ethical, cultural, and scientific considerations specific to inclusivity in global research is included in the Supporting Information

Specimen numbers for bones: EP19.1_F101 - F167, EP19.1_F201 – F208, EP19.1_F301 – F327, EP19.61_F101 – F179, EP19.8_F101 – F199, EP19.8_F201 - F266, EP19.8_F301 – F365.

Specimen numbers for lithics: EP19.5_L1 - EP19.5_L17, EP19.86_L1 - EP19.86_L181, EP19.87_L1 - EP19.87_L33, EP19.1_L1 – EP19.1_L445, EP19.83_L1 - EP19.81_L10, EP19.10_L1- EP19.10_L10, EP19.11_L1 - EP19.11_L15, EP19.13_L1 - EP19.13_L6.

## Results

### Landscape and hydrology of the As Sulb plateau

Our team focussed our fieldwork on an area measuring approximately 10 x 10 km, located just north of the village of Shawyah (Fig 1). We hypothesise that in the Palaeocene-Early Eocene the region around the caves was a coastal embayment that was periodically cut off from the sea, leading to evaporative concentration and gypsum precipitation. This would explain why the caves are concentrated in a small area of a much more extensive shallow marine limestone outcrop. It has been suggested that karstification of the Umm er Radhuma Formation started in the Oligocene, prior to the deposition of the overlying Miocene-Pliocene units [60].

Palaeohydrological mapping shows that the area is scattered with small palaeolakes fed by local palaeoriver systems (Fig 1). To the southwest of the As Sulb plateau lies the Ad-Dahnā Sand Sea that in contrast lacks mapped palaeohydrology. However, in the northeast of the study area the PALSAR HH radar imagery shows a complex network of river channels emerging from under the dunes (Figs 2 and 3). Some of these channels are contained in shallow valleys cut into the surface geology, while others exhibit inverted topography. The latter are ancient channels where the river gravels are more resistant to erosion than the underlying limestones and thus have protected them from aeolian erosion. Over time this led to the channels forming long sinuous ridges. On the western side of the Ad-Dahnā a large palaeoriver system can be seen flowing to its western edge and then abutting against the dunes where it forms a playa at Rawdath Tinhat (Fig 2). Previously this river system would have continued to the north-east, as indicated by the complex of channels that are observed on the eastern side of the Ad-Dahnā.

**Fig 4:**
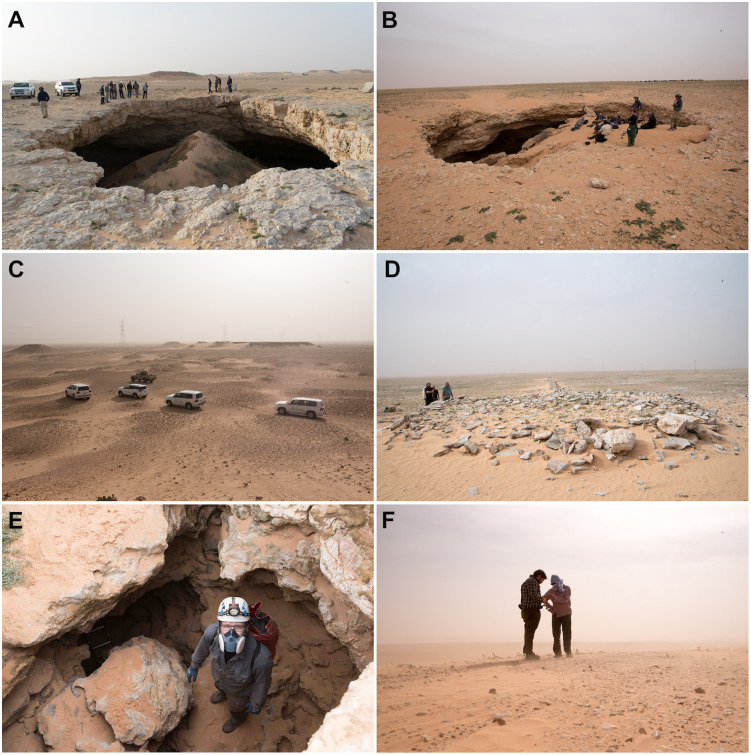
Landscapes, caves, and archaeology of Northeast Arabia. A: Abu Jirfan, a large vertical cave opening, B: the entrance of Murubbeh Cave, C: general view of As Sulb landscape, D: EP19.90 pendant tomb, E: the entrance to Mossy Cave, F: EP19.10, a fluvial gravel deposit associated with lithics.

**Fig 5:**
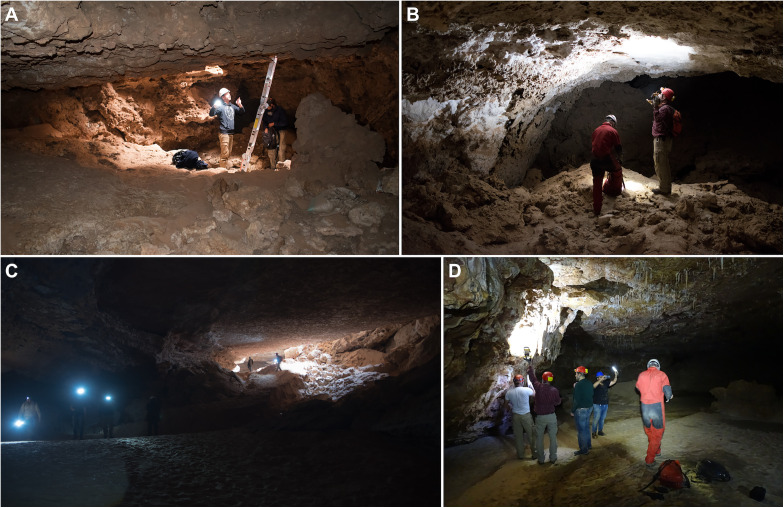
Cave passages in Murubbeh Cave (B, C) and EP19.8 (A, D).

Most of the investigated caves have vertical openings, with entrance shafts between five and 15 metres deep. In many cases the bases of these shafts are blocked with rocks and sand. In other cases, relatively extensive systems of horizontal passages can be accessed. The vertical nature of most cave entrances in the area (Fig 4) often makes access rather challenging. In some cases, the caves can be accessed on foot, but this is typically difficult. Murubbeh Cave (Figs 4 and 5), for instance, primarily consists of a steeply sloping, but not vertical, passage down to a large chamber. From an archaeological perspective, few of the caves would seem to present attractive locations for human occupation, and indeed aside from occasional lithics around the entrances of a few sites, we did not identify any significant archaeological potential in most of the caves.

A significant finding of our research related to the hydrology of the area. Our fieldwork in January 2019 took place just after a large storm had impacted the area in November 2018. This led to very heavy rainfall, and indeed in nearby Kuwait, this was described by a regional newspaper (*The National*) as a “once-in-a-lifetime storm” (https://www.thenationalnews.com/world/mena/jeddah-storm-warning-as-deadly-floods-sweep-across-saudi-arabia-1.792074). Subsequent research identified 2018 as a particularly wet year [61]. It became clear during our visit that large amounts of water, and sediments, had entered the caves. For instance, Kahf al Rutuwbah is a previously reported horizontal cave system [51], but at the time of our survey was filled with sand, making access impossible. Likewise, Surprise and Friendly Caves, also previously reported [e.g., 62] appear to have been buried by sand relating to these rains. At another location, named Hotel Cave by the Saudi Geological Survey, large banks of sediment had been eroded away and washed into the cave. One previously walking-height passage was filled almost to the roof. In other cases, the rain had flushed sediment out, such as in the case of Mossy Cave, which previously contained much sediment, but did not when we visited. Given the rather flat nature of the area, and hence limited catchments for individual caves, these findings indicate the extreme nature of the rainfall which had occurred. These findings highlight the extent to which high-intensity storms can lead to large amounts of precipitation, which clearly leads to extensive aquifer recharge. These hydrological characteristics also mean that it is likely that palaeontological and archaeological materials have been transported by flood activity.

### Palaeontology of the caves

#### Murubbeh Cave.

**Fig 6:**
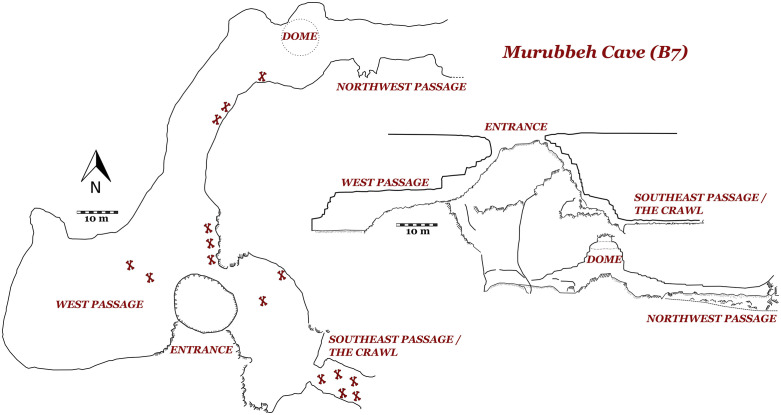
Plan and section views of Murrubeh Cave. The location of significant bone accumulations is shown. Map based on [48].

Murubbeh Cave (also known as B7 Cave and additionally given the code EP19.61) comprises a small doline (Fig 4) from which three passages descend underground [52,62] (Fig 6). The most accessible of these are the ‘west’ and ‘northwest’ passages, which have been the focus of previous preliminary work. The west passage quickly opens into a large sandy chamber with no visible macrofauna remains, but abundant microfauna (reptiles, rodents, birds, and bats) situated near the entrance and likely beneath a bird roost, and similar deposits were observed near the entrance to the northwest passage. Following an initial steep descent, the northwest passage flattens into a lengthy horizontal channel containing thousands of animal bones, predominately camel but also including equids, gazelles, and carnivores. Similarly, the northeast passage initially descends steeply, before opening into large chamber, again containing thousands of large mammal bones. Finally, we observed a very low but wide and sprawling flat opening (dubbed “the crawl”) where perhaps tens-of-thousands of large mammal bones were scattered.

**Fig 7:**
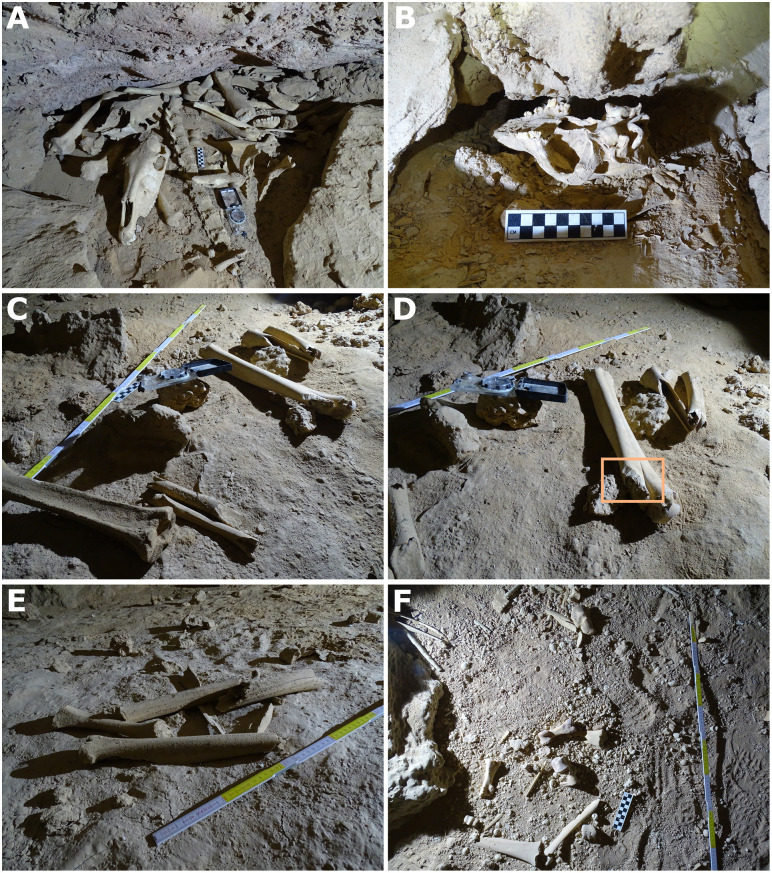
Examples of bone assemblages from caves of Northeast Arabia. (A) Tightly packed bone assemblage within a wall-floor crevice, northeast passage of Murubbeh Cave; (B) wolf/dog skull, EP19.8; (C-E) EP19.91 cache 3 bone assemblage showing the aligned orientation and an example of porcupine gnawing (orange box); (F) EP19.91 cache 1 bone assemblage with several gnawed camel bones.

These large bone accumulations appear to be the product of various biotic and abiotic factors. Preliminary on-site analysis of one such assemblage (“Cache 1”) in the northeast passage (Fig 7A) suggested a carnivore-accumulated bone cache, given the density of material and extensive carnivore gnawing, similar to known hyena dens in Saudi Arabia [9]. However, the removal of the surface material revealed the assemblage to be tightly packed into an inaccessible floor-wall crevice, suggesting that the material had redeposited during flooding Similar deposits can be observed throughout the cave, while others such as those positioned at certain high points, appear to represent largely intact carnivore caches. Recent visitors have also moved bones around the cave, evident in stacks of bones (e.g., wolf skulls) neatly placed atop boulders. Such disturbances were not noted in the more inaccessible southeast passage, likely owing to its difficulty of access.

**Fig 8:**
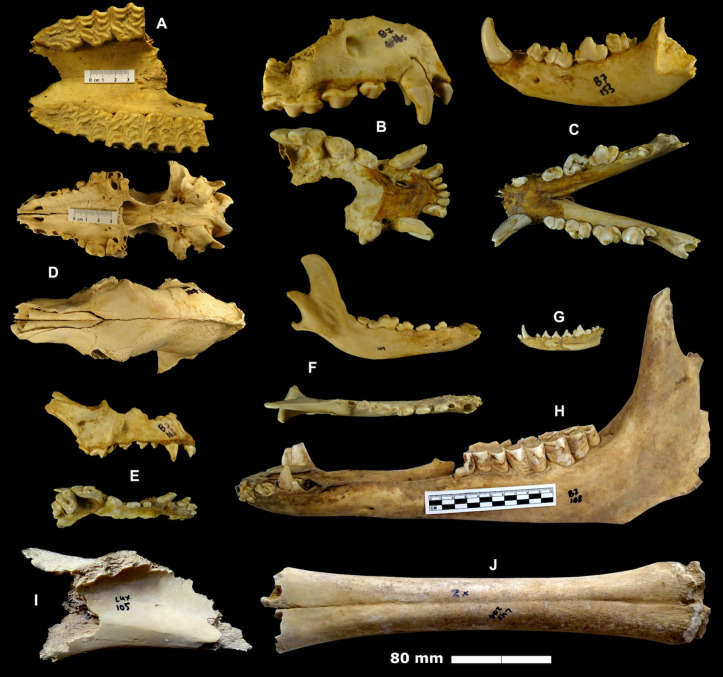
Macromammal remains recovered from the caves. A-H: Murubbeh, I-J: EP19.91. Equid maxilla (A), striped hyenas (*hyaena hyaena*) maxilla (B) and mandible (C); canid (*Canis* sp.) crania (D, E) and mandible (F); red fox (*Vulpes vulpes*) mandible (G); camel (*Camelus dromedarius*) mandible (H); and examples of a heavily gnawed large ungulate scapula (I) and camel metapodial (J).

**Fig 9:**
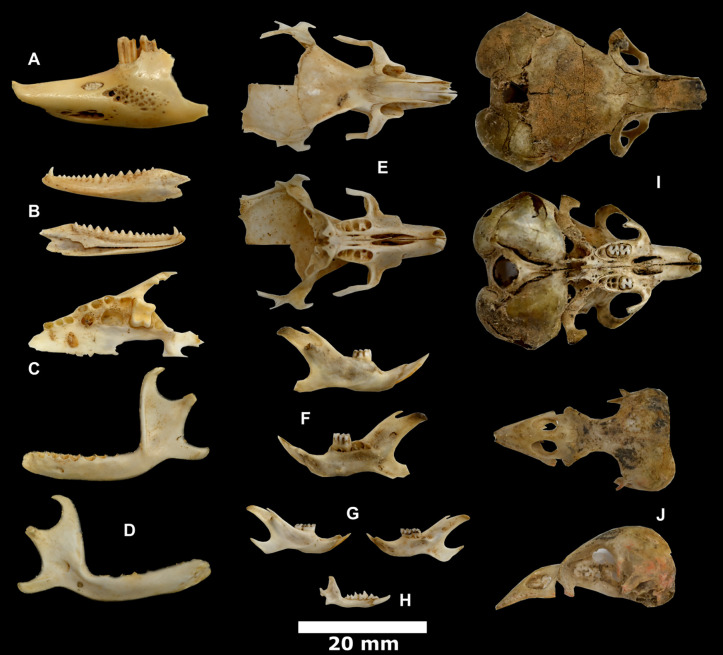
Microfauna remains recovered from Murubbeh Cave. Cape hare (*Lepus capensis*) mandible (A); spiny-tailed lizard (*Uromastyx* sp.) mandible (B); cf. desert hedgehog (cf. *Paraechinus aethiopicus*) maxilla (C) and mandible (D); lesser Egyptian (*Gerbillus gerbillus*) or Cheesman’s gerbil (*G. cheesmani*) crania (E) and mandible (F); Balochistan (*G. nanus*) or Wagner’s gerbil (*G. dasyurus*) mandible (G); shrew (*Suncus* or *Crocidura*) mandible (H); lesser Egyptian jerboa (*Jaculus jaculus*) crania (I); and perching bird (*Passeriformes*) crania (J).

Bone and other organic preservation at Murubbeh Cave is remarkable, exemplified by the earlier discovery of a 2,000-year-old mummified fox [62]. This is likely due to the dry conditions within the cave, and the low and consistent temperature which sits at around 18 ^0^C year-round [62]. Species identified include a variety of domestic (e.g., camel, cattle, sheep, goat) and wild (e.g., oryx) ungulates, small (e.g., sand cat, red fox) and medium-sized (e.g., wolves, dogs, striped hyena) carnivores, and a variety of microfauna including rodents (e.g., gerbils), reptiles (e.g., spiny-tailed lizards), birds, and bats (Table 1) (Figs 8 and 9). Hyena and wolf coprolites are also abundant throughout the cave. Two human skulls, as well as leather, including one large and neatly folded piece the size of a “small blanket”, have also been previously reported at Murubbeh Cave [62]. Further studies using methods such as sieving and flotation would be likely to significantly boost the recovery of small faunal remains from the site.

**Table 1:** Taxonomic list from (1) Murubbeh, (2) EP19.8, and (3) EP19.91 caves. For completeness, species described by Bauer [52] and Pint [50] from Murubbeh have been included. Species highlighted in bold were identified previously and not as part of the present study. Note, numbers 1-3 in the first refer to Murubbeh, EP19.8, and EP19.91 caves respectively. X’s represent the presence of this species at each site.

Class	Order	Family	Taxon	Common Name	1	2	3
Reptilia							
	Squamata	Agamidae	*Uromastyx* sp.	Spiny-tailed lizard	X		
Aves							
	Columbiformes	Columbidae		Dove/ pigeon	X		
	Passeriformes			Perching bird	X		
Mammalia							
	Artiodactyla	Bovidae	*Bos taurus*	Cattle	X		
			*Capra/Ovis* sp.	Ovicaprid	X	X	
			*Oryx leucoryx*	Arabian oryx	X		
			*Gazella dorcas*	Dorcas gazelle	X	X	
			*Gazella marica*	Arabian sand gazelle	X		
			***Gazella subguturrosa***	**Goitered gazelle**	**X**		
			*Gazella* sp.	Gazelle			X
		Camelidae	*Camelus dromedarius*	Dromedary camel	X	X	X
	Perrisodactyla	Equidae	*Equus* sp.	Horse/ donkey	X	X	X
	Carnivora	Canidae	*Canis lupus*	Wolf/ dog	X		X
			*Vulpes vulpes arabica*	Arabian red fox	X		X
			*Vulpes rueppellii*	Rüppell’s fox.	X		
		Hyaenidae	*Hyaena hyaena*	Stripped hyena	X		
		**Felinae**	***Felis margarita***	**Sand cat**			
	Eulipotyphla	Erinaceidae	cf. *Paraechinus aethiopicus*	Desert hedgehog	X		
		Soricidae	*Suncus/ Crocidura*	Shrew	X		
	Lagomorpha	Leporidae	*Lepus capensis*	Cape hare	X		
	Rodentia	Muridae	*Merionus* sp. cf. *M. libycus*	Libyan jird	X		
			*Gerbillus gerbillus/ cheesmani*	Lesser Egyptian gerbil/ Cheesman’s gerbil	X		
			*Gerbillus nanus/ dasyurus*	Balochistan gerbil/ Wagner’s gerbil	X		
		Dipodidae	*Jaculus jaculus*	Lesser Egyptian jerboa	X		
		Hystricidae	*Hystrix indica*	Indian crested porcupine	X		
	Primates	Hominidae	*Homo sapiens*	Human	X		
	Chiroptera			Bat		X	

#### Cave EP19.8.

Like many in the area, this cave begins with a short vertical drop leading to a complex network of horizontally sprawling passages (Fig 5). Despite investigating the cave for several hours, it is likely that much of it remains unexplored. Passages were typically very narrow and movement in the cave was often restricted. Stalagmites and stalactites were found throughout the cave, and chert nodules were visible in the roof in places. Recent flooding was evidenced by wet sand and water marks on the cave walls.

**Fig 10:**
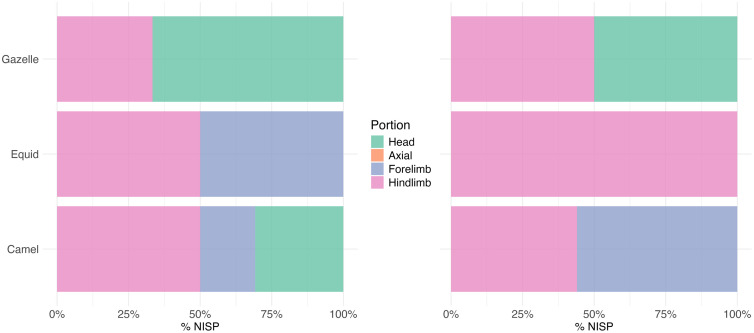
NISP by body portion for gazelle, equid, and camel at EP19.8 (left) and EP19.91 (right).

As at Murubbeh Cave, hundreds of exceptionally preserved bones were found, mostly concentrated on high areas protected from flooding—either large sand mounds, boulders, or areas of rock fall. Three distinct assemblages (caches 1–3) were targeted for a pilot analysis though as above no sieving was conducted and it is likely that the assemblages are biased toward larger, more easily spotted remains in dim light. Considering this, as well as the small sample sizes, we combine here the three assemblages and report the findings as the total number of identified specimens (NISP) (Fig 10). The breakdown of NISP by cache can be found in Appendix 1 in S1 Data.

A total of 224 bones were examined from across three caches. Unidentifiable midshaft fragments of small (e.g., gazelles), medium (e.g., ovicaprid), and large-sized (e.g., camels) mammals make up the bulk of the assemblages (n = 138, 62%). Camel is the best represented (NISP = 37), followed by gazelle (NISP = 22), equid (NISP = 8), and a small number of hyena, carnivore, and microfauna remains including reptiles and bats (Table 1). Camels and equids were primarily represented by limb elements, and in particular dense elements like metapodials and astragali (Fig 10). In contrast, gazelle was best represented by elements of the crania, including horn cores, maxillae, and mandibles. In addition, a scapula, radius, and two humeri of a medium-sized bovid, possibly a sheep or goat, were recovered from cache 3.

Preservation ranged from fresh to heavily stained (brown) and corroded, with many large mammal teeth powdery and fragile, likely the result of water damage. The small-and-medium sized animal remains in Cache 2 indicate fluvial transport deep into the cave. Extensive gnawing of several bones suggests initial carnivore accumulation, with later redeposition by floodwaters Additionally, some remains may have entered the cave via porcupines, as indicated by distinctive chisel-like gnawing marks on a few specimens (Fig 7D), including a camel metapodial and two equid proximal phalanges.

#### Cave EP19.91.

Entrance into this cave requires vertical descent of approximately eight metres. The cave includes numerous sprawling passages, many of which are tall, sandy-floored, and generally easy to navigate. Despite prolonged exploration of this cave, its extensive nature means that it is probable that there are parts that were not discovered. The most impressive sections of the cave are two very large chambers, approximately 30 x 15 m in plan. The first of these is particularly striking, containing abundant speleothem formations, including curtains, stalactites, and less commonly, stalagmites.

The second chamber, which was located a short crawl from the first, contains abundant animal remains. Again, bones from three distinct accumulations were examined in a pilot study. Cache 1 was situated atop a high mud/rock fall mound and although the sediment was wet and fossils slightly water damaged it appears that it may have been protected from the most severe effects of flooding. Caches 2 and 3 were clearly redeposited during the recent flooding event, as indicated by the aligned orientations of many of the bones (Fig 7C and 7D).

A total of 71 bones were examined from across the three caches. Again, unidentifiable long bone fragments of small, medium, and large-sized mammals are abundant (n = 20, 41%). The bulk of the rest of the material comprises camel (NISP = 34), consisting entirely of hindlimb and forelimb elements (Fig 10). A single equid femur and a gazelle horn core and tibia were also recovered, as well as a few small carnivore and medium-sized bovid remains. As before, many of the bone exhibited extensive gnawing, indicating accumulation by carnivores. Also, juvenile camel remains were present in all three caches, likely selectively targeted by carnivores on the landscape. The discovery of fresh fox prints, and that of a relatively fresh fox carcass in the chamber, indicate that foxes currently reside in the cave.

To gain an understanding of the chronology of bone accumulations, five teeth were selected for radiocarbon dating; three from Murubbeh Cave and two from EP19.8 (Table 2). Some of the bones are relatively recent, with two equid teeth from Murubbeh dating to approximately 650 and 200 years cal. BP. There is, however, some temporal depth to the material, with a cattle tooth from Murubbeh producing an age of approximately 3300 years cal. BP. The samples from EP19.8 produced ages ranging between approximately 1000–2000 years cal. BP. These samples indicate that the observed bone accumulations date primarily to the Late Holocene.

**Table 2:** AMS radiocarbon ages from the Murubbeh and EP19.18 caves. Samples were calibrated using Calib (v.8.20), with IntCal20 [63]. The *Vulpes* radiocarbon date is from Forti et al. [62].

Site	Lab code	Taxon	Radiocarbon age (years bp)	Error (years)	Cal. BP (2 sigma)	Median Age
Murubbeh	GU55742	*Equus*	716	26	685–570	667
Murubbeh	GU55744	*Equus*	169	26	288–0	178
Murubbeh	GU55743	*Bos*	3064	31	3363–3177	3281
Murubbeh	ETH-25068	*Vulpes*	1890	45	1924–1709	1799
EP19.8	GU55746	*Camelus*	2029	30	2095–1881	1965
EP19.8	GU55747	*Camelus*	1049	20	1053–917	948

In terms of the chronology of the bone accumulations, with the caveat that only a small number of bones from a large number have been dated, their relatively recent age is noteworthy. The overabundance of livestock remains may indicate an intensification of human activity in the landscape during the Holocene, coinciding with the spread of camels, cattle, ovicaprids and equids to the area. It is possible that older fauna are buried in deposits in these caves, and/or have been removed by flooding.

### The archaeology of the As Sulb Plateau

Remote sensing data was used to target different landscape features in the As Sulb Plateau. While the surface in some areas is covered in aeolian sand, large exposures of bedrock and gravel deposits in places mean excellent visibility of archaeological features. Chert outcrops are common in the area, and lithic assemblages are often associated with them. In most cases lithic assemblages are very low density, with the exception of one locality, EP19.1, which is discussed separately below. The key new archaeological sites are indicated on Fig 1.

**Fig 11:**
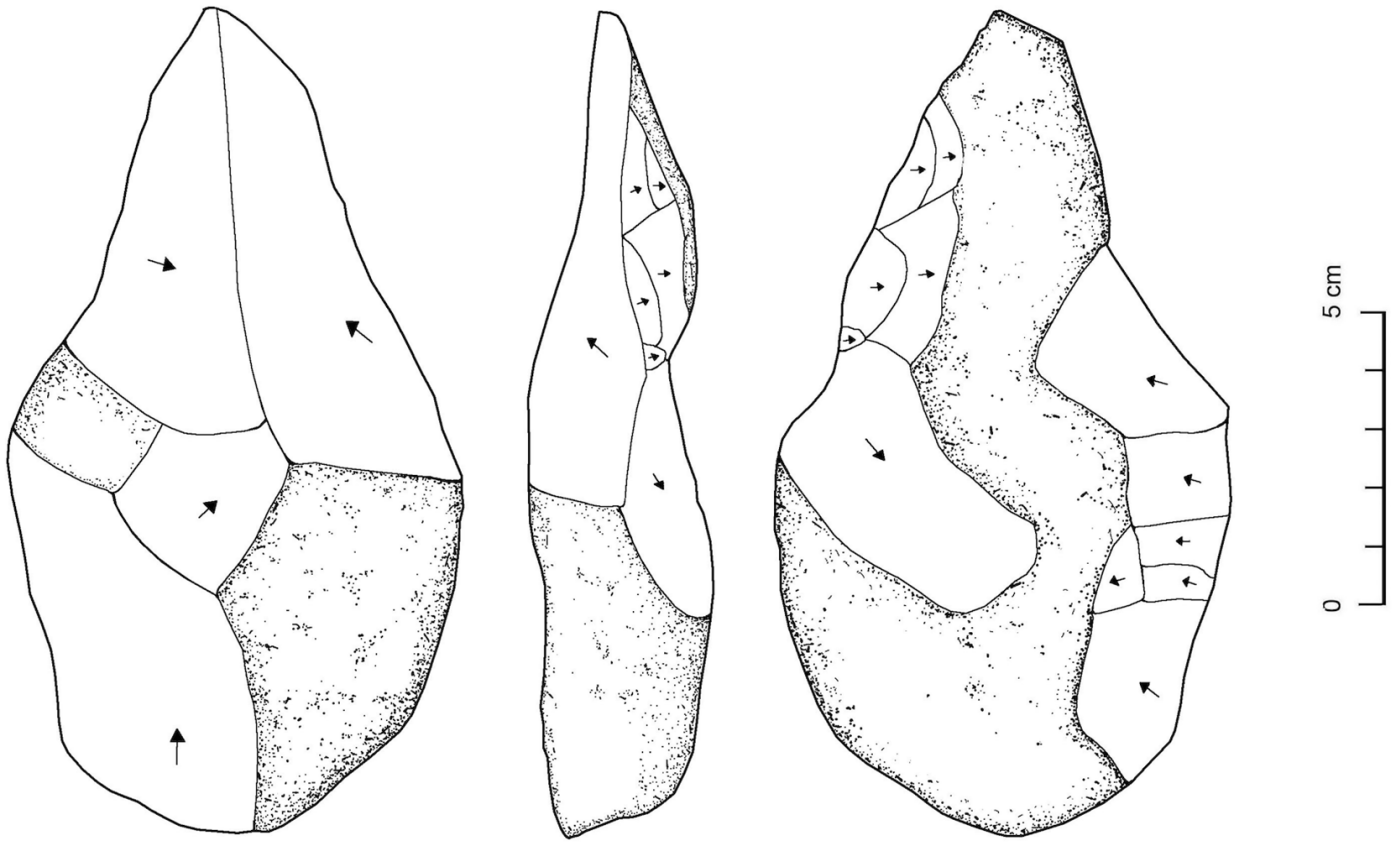
Chert handaxe from EP19.5. Arrows indicating direction of flake removals. In this, and subsequent figures, the hatched areas show cortex.

At locality EP19.5 eroding chert was exposed over a wide area. In one area, where large chert nodules were exposed, a small collection of artefacts was noted. This consisted of 11 flakes, 5 cores indicating simple flake production, and a single handaxe (Fig 11). The handaxe is 14 cm long, and was crudely flaked, with cortex remaining on both surfaces. The handaxe, and associated crude core and flake technology, are consistent with a Lower Palaeolithic attribution.

Site EP19.86 is located next to a deep vertical cave which may have provided a water source. Stone structures were identified, including the remains of a small settlement with several hearths, some Islamic graves, a large circle of stones, an ‘open air mosque’ (sometimes also called ‘desert mosques’) and a few ceramics, representing the only archaeology of the recent past that we observed in the area. Lithics were also present at the site, where chert outcrops occur. A total of 23 cores and 58 flakes were collected to characterise the assemblage. The most common core forms are single platform (nine) and multiplatform cores (six). Four of the cores are Levallois cores; two recurrent centripetal and two preferential with centripetal preparation. Some of the cores clearly make use of naturally ‘Levallois-like’ convexities. Thirteen retouched flakes were collected, mostly ca. 30–60 mm in length. These are of varied blank form and retouch character, but mostly different forms of ‘scraper’-like retouch. The lithics from EP19.86 may represent more than one period, and are mostly rather simple and non-diagnostic, but overall are of a broadly Middle Palaeolithic character.

**Fig 12:**
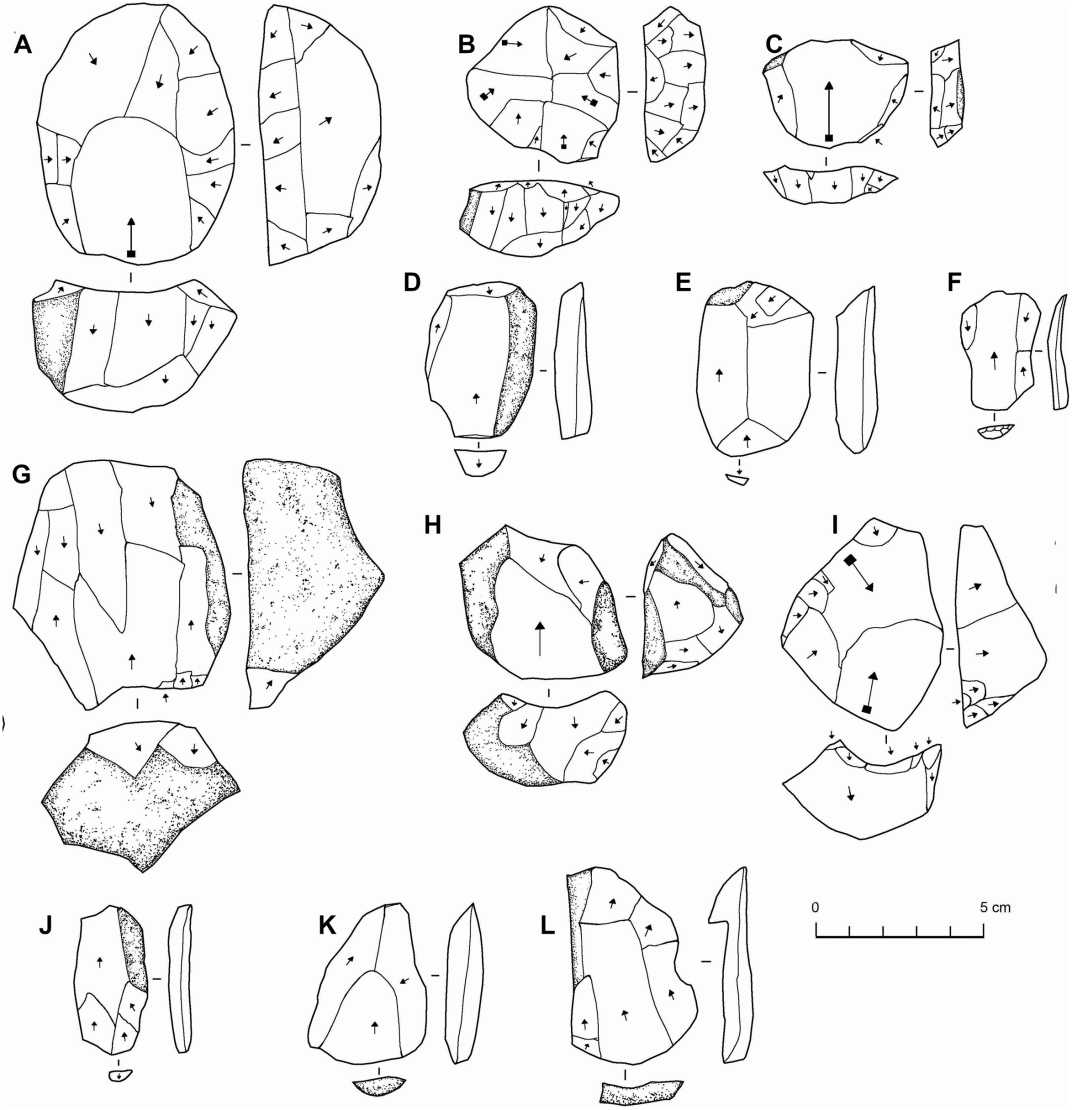
Lithics from EP19.86 (A-F) and EP19.87 (G-L). Cores (A-C, G-I) and flakes (D-F, J-L). All are chert. These illustrations, and those in subsequent figures, highlight the basic shapes of these artefacts, with the arrows indicating the directions of flake removals. Arrows with a square symbol at their proximal end show removals interpreted as Levallois removals.

Site EP19.87, located close to EP19.1 and EP19.86, represents a chert covered hilltop, the largest outcrop observed in the area. A low-density scatter of stone tools was identified. As well as mostly rather non-diagnostic flakes, varied cores are present. A representative sample of 20 cores and 13 flakes was collected in the densest area of the site. The most common forms are single platform and multidirectional cores (six of each), with other forms including three bidirectional cores and a single orthogonal core. The cores are mostly ca. 60–80 mm in length, 40–60 mm in width, and 30–50 mm thick. The assemblage has a Middle Palaeolithic character, though rather simple in reduction technology, and somewhat varied. Levallois and Levallois-like cores are present in the assemblage (Fig 12), but in low numbers. It is evident in many cases that clasts with a natural Levallois-like morphology, such as a natural hierarchy of surfaces, have been selected for flaking. In most cases the cores show relatively few removals, which are typically flake removals, though in a few cases more laminar removals are evident. There is little evidence for careful platform preparation such as faceting. Among the flakes, some have the morphology of Levallois flakes though with little platform preparation. The lithics display variable amounts of weathering, with some chert examples showing seemingly desilicified surfaces, perhaps indicating a temporal depth to their production. In some cases, there is a clear double patina, with, for instance, one flake being worked into a notch, with the notched area much less weathered than the rest.

Site EP19.90, which was identified via satellite imagery, is a large pendant tomb. In contrast to some areas of Arabia where these forms are abundant and are attributed to the Bronze Age [64], this tomb was the only such example that we found in the area. Indeed, the paucity of stone structures and of hearths is a striking feature of the study area. EP19.90 is located in an area of (relatively) high ground. It features a large central cairn (Fig 4), around 10 metres across, with some well-built dry-stone walling visible in places. At the centre of the cairn, looting had revealed some loose bones. One was selected for radiocarbon dating, though age determination was not successful. A long ‘tail’ extends away from the cairn to the northwest, for 135 metres. The tail varies along its length, consisting in places of small cairns, ‘keyhole’ shaped structures in others, and a small stone circle. No other finds, such as lithics or pottery, were found around the pendant.

#### The EP19.1 lithic assemblage.

In contrast to the low-density lithic artefact scatters found in the surveys, one locality (EP19.1) recorded a moderately dense distribution of lithics. The surface lithics were systematically collected, and consisted of 445 artefacts from an area measuring approximately 40 x 40 m. The site is on a slight rise on the edge of a group of hillocks which form a fairly prominent feature in this generally flat landscape. Chert can be seen eroding from the ground. The site is immediately adjacent to a deep (ca. 20 m) cave opening. A local Bedouin shepherd informant noted that his grandfather used to climb down into the cave using a rope to collect water. It is likely that during times of increased rainfall and a higher water table that this would have made this water source easier to access.

The lithic finds at EP19.1 were almost all chert, presumably mostly derived from the outcropping material on site. The character of the chert used for lithic manufacture varied, with some more silicious than others, and with somewhat variable weathering, although most of the assemblage had a similar, moderate, weathering. The only notable raw material diversity consists of two cores being made on quartz, likely sourced from fluvial gravels which occur in the region.

The lithic assemblage is dominated by flakes, which make up 69% of the collection (Table 3). Both cores and retouched flakes are well represented. In addition, one probable hammerstone was recovered. As summarised using mass (in grams) in Table 3, the EP19.1 assemblage is characterised by small-sized lithics.

**Table 3:** Summary of the EP19.1 lithic assemblage.

	Cores	Flakes	Retouched
**n.**	98	315	41
**Mean weight (g)**	25.8	9.5	16.9
**25% weight (g)**	20.0	3.5	9.1
**75% weight (g)**	43.8	12.6	22.1
**s.d.**	26.2	9.4	10.8

**Fig 13:**
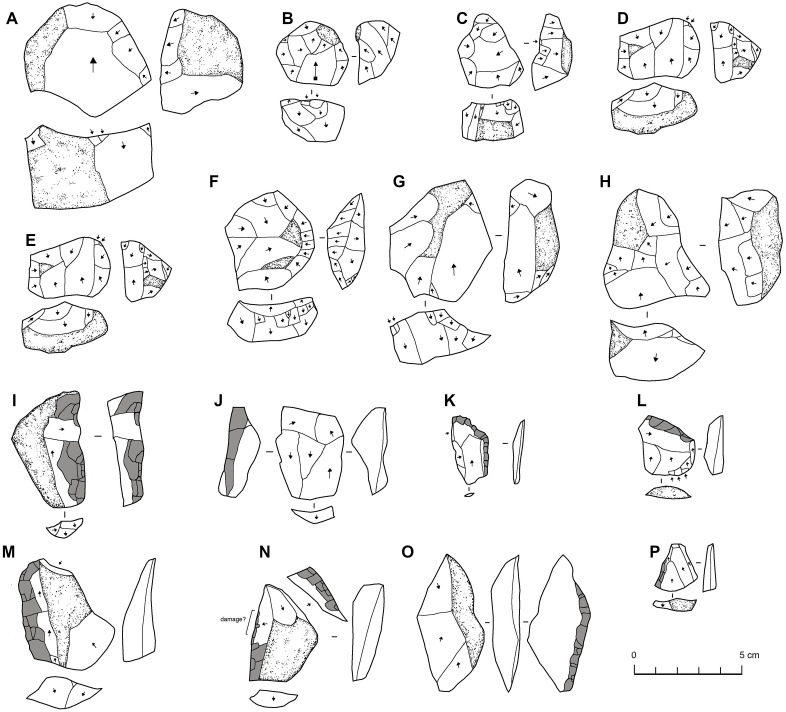
Cores (A-H) and retouched flakes (I-P) from EP 19.1. All retouched tools are ‘scrapers’ except the second example which is a burin. All artefacts are chert.

The typology of the cores is summarised in Table 4 and illustrated examples are shown in Fig 13. Most cores retain cortex, which indicates relatively limited reduction of small clasts, although in some cases there are a relatively large number of small flake scars relative to core size. The EP19.1 cores are relatively diverse. Single platform cores, with just a few flakes removed, and multiplatform cores, typically with several distinct debitage surfaces, are the most common forms. Some with a simple centripetal reduction are classed as radial cores. Some of the cores can be classified as Levallois cores, typically preferential with centripetal preparation. Some are Levallois-like, with varying combinations of ‘typical’ Levallois features. For instance, some show more extensive flaking on both surfaces of the core than is typical in Levallois where there is a rigid separation between preparation and exploitation surfaces.

**Table 4:** Core typology and weight of the EP19.1 cores.

Type	Number	Percent	Mean weight (g)
Single platform	21	21.4	44.7
Radial	12	12.2	46.4
Multiplatform	25	25.6	33
Core on flake	6	6.1	19.6
Levallois-like	12	12.2	45.9
Levallois preferential	10	10.2	22.4
Levallois recurrent	1	1.0	7.4
Triangular	6	6.1	38.7
Core frag./indet.	5	5.1	11.2

There are also forms that we label ‘triangular’ cores, which are Levallois-like in many regards, but the debitage surface has two exploitation surfaces angled away from each other, giving the core a triangular cross section, rather than the single, basically flat, surface associated with Levallois *sensu stricto*. It could be that these are Levallois preforms/unstruck Levallois cores in some cases. Beyond typology, the key characteristic of the assemblage is that it shows an emphasis on flake production, with seemingly hard hammer reduction of relatively small cores. Where there are more elongate removals, they seem to be debordants. While hard to quantify, the cores are often reminiscent of Levallois cores in their morphology, but there is a focus on removing multiple small flakes instead of a smaller number of relatively large (Levallois) flakes. The cores show some mild platform preparation (faceting), but it is not particularly extensive.

**Fig 14:**
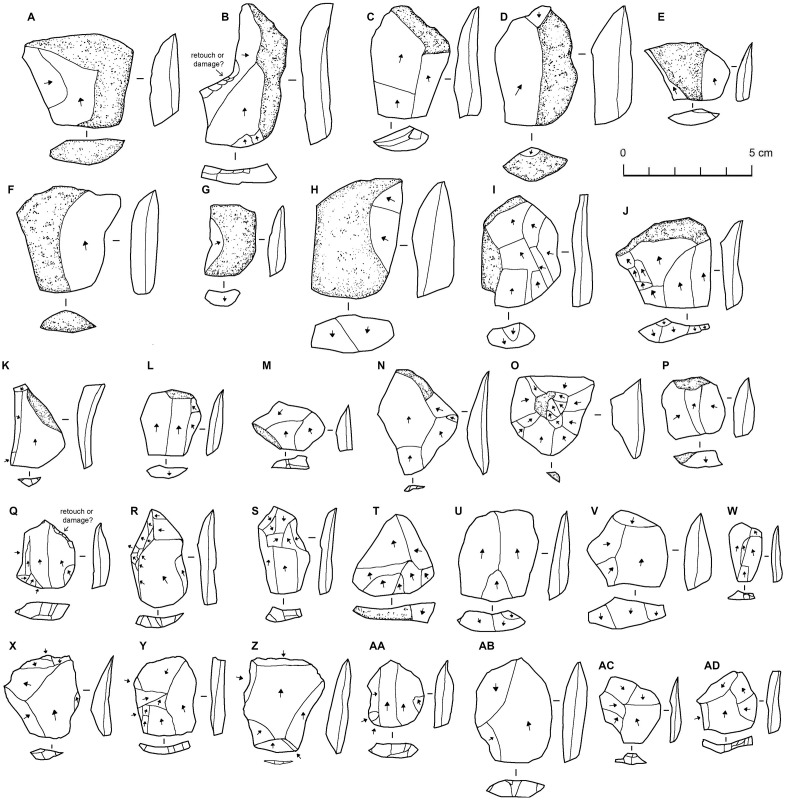
Chert flakes from EP19.1. Flakes at the top with more cortex are interpreted as probably coming from the earlier stages of reduction, while those at the bottom are the result of the later stages of reduction.

Most flakes are small (mostly under 10 grams) and relatively squat, and many have cortex on their dorsal surfaces (Fig 14). While the bulbs are typically somewhat diffuse, they nevertheless suggest hard hammer percussion. While many flakes have plain or cortical platforms, prepared platforms are common, and in some cases, they are fairly finely facetted. A small number (n = 11) of the flakes can be described as Levallois flakes, based on their shape and character of dorsal surface preparation. They are all basically parallel sided in shape, and almost all have centripetal or sub-centripetal scar patterns. Elongate flakes are rare, and where present seem to be core management elements rather than the result of deliberate blade/bladelet production.

A relatively large sample of retouched artefacts (compared to most Arabian sites) was recovered from EP19.1. While there is some variation in how these can be classified (Table 5), such as one burin, for the most part the retouch presents a basically consistent character. The blank characteristics appear comparable to the general flake population, with just slightly larger and thicker flakes being selected. The retouch is typically rather coarse, sometimes grading into notching, and was generally only applied to the dorsal surface. The retouched tools reflect different combinations of lateral and distal retouch (Table 5). The retouch is typically rather steep ‘scraper’-like retouch. A single example has clearly younger (much less weathered) retouch, along the lateral margin of the ventral surface of an older flake.

**Table 5:** Summary of typology and weight of retouched tools from EP19.1.

Type	Number	Percent	Mean weight (g)
Side retouched	5	12.2	18.3
Double side retouched	3	7.3	11.1
Side and end retouched	8	19.5	20.6
Double side and end retouched	9	22.0	14.6
End retouched	5	12.2	17.3
Notched, side, and end retouched	1	2.4	37.9
Notched, double side, and end retouched	3	7.3	10.1
Denticulated, double side retouched	1	2.4	9.3
Bifacially flaked piece	2	4.9	5.9
Double burin	1	2.4	22.2
Retouched plaquette	1	2.4	7.8
Broken/indet.	2	4.9	32.2

No suitable material for chronometric dating was available at EP19.1. Therefore, chronological and cultural attribution relies on the characteristics of the material culture. As well as the single example of a retouched flake with double patina and a few seemingly fresher flakes, seven small fragments of pottery were recovered from the site. All but one are rather thin (<15 mm), and most show clear evidence of wheel spinning. For the most part, they have an orangish interior and fabric, and a green exterior, clearly green slip in some cases. Two pieces have linear decoration, while another two have triangular-like indentions. Given the presence of pottery, one interpretation is that the EP19.1 assemblage is therefore Holocene in age. However, given the dominant techno-typological characteristics of the lithic assemblage, such as the use of the Levallois method, widespread striking platform faceting, and simple ‘scraper’-like retouch, indicating that the assemblage is Middle Palaeolithic, with a minor subsequent re-use of the site. We suggest the distinctive characteristics of the assemblage – Levallois-like core shapes but with a more amorphous, multiplatform-like, reduction technology, and a relatively high frequency of retouched flakes, at least partly reflects raw material factors, with knappers adapting to small clast size. Minor re-use of the site, as shown by the pottery, is not surprising given the availability of water and chert at the site. Common indications of Holocene sites in the region, such as arrowheads, grinding stones, hearths, and stone structures are conspicuously absent.

#### Archaeology of the palaeorivers and palaeolakes.

**Fig 15:**
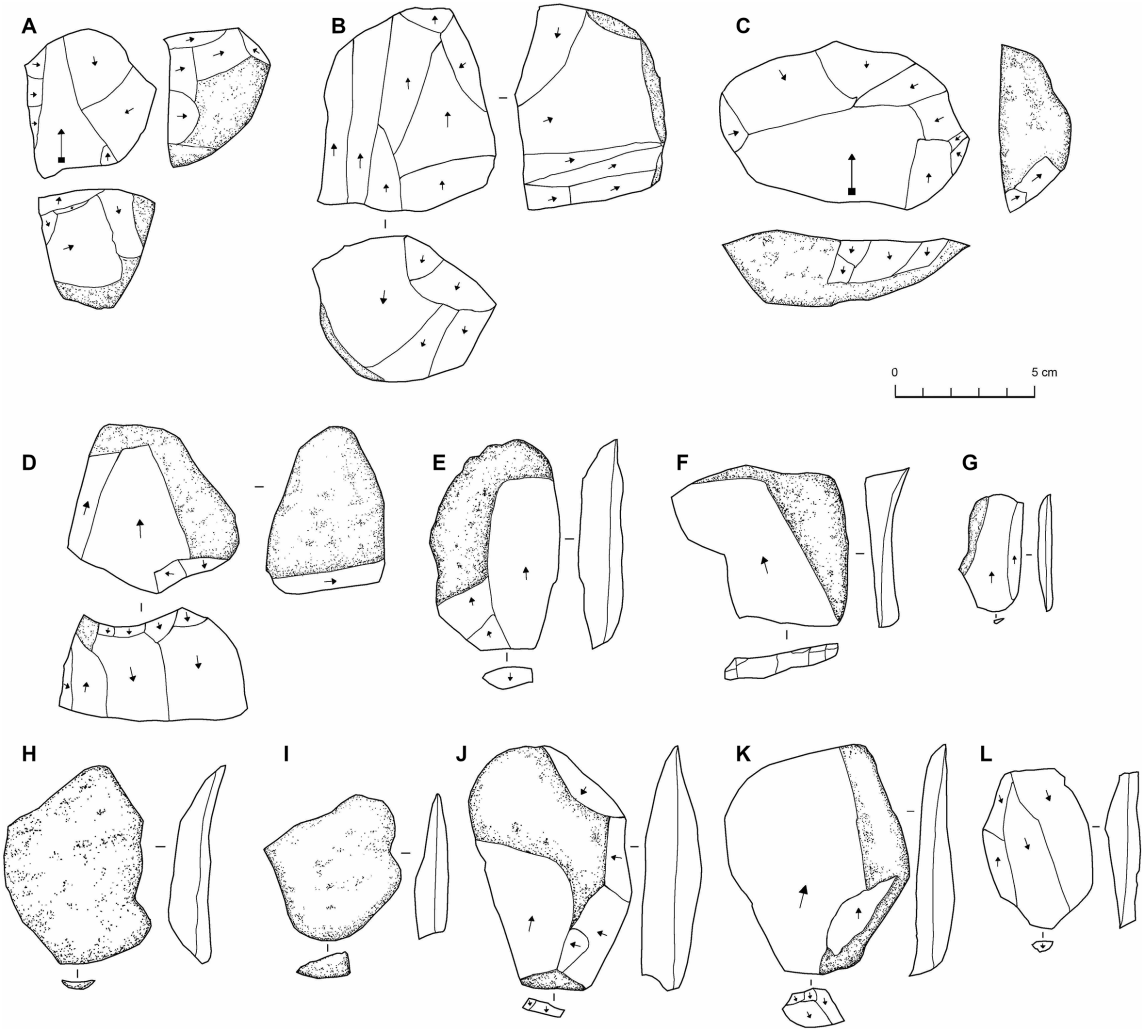
Lithics associated with northeast Arabian palaeorivers. From sites EP19.10 (B, H-L), EP19.11 (A, E-G), EP19.13 (C,D), and EP19.83 (B). Cores: A-D, flakes: E-L.

Surveys were conducted along a large palaeolake on the western side of the Ad Dahnā sand sea and several palaeorivers evident in the PALSAR radar imagery which occur around 40 kilometres northeast of the As Sulb karstic plateau (Figs 2 and 3). No archaeology was evident in the vicinity of the Rawdath Tinhat palaeolake. This is of playa form and appears to experience frequent flooding even today and therefore any archaeology may be buried under recent sediments. With respect to the rivers, many took the form of inverted relief features, where the gravel deposits of the rivers provide an armoured surface more resistant to erosion than the surrounding landscape. The gravels consisted of diverse lithologies and reflect ancient drainage patterns from central Arabia eastwards. In places these gravel deposits are extensive. In all examples visited, only very low-density distributions of lithics were recorded (Fig 15).

At site EP19.83 three cores and seven flakes were recovered (Fig 15). These have diverse weathering and technological characteristics, and so parsimoniously reflect occasional use of the site as a raw material source. While some examples are Middle Palaeolithic-like, the example shown in Fig 15 had a focus on laminar removals and may be Late Palaeolithic or Neolithic. Site EP19.10 supports the observation that the lithics associated with the gravels are not the result of transport, but rather of subsequent use of the gravel deposits as raw material sources. Across an area measuring approximately 150 metres, only occasional flakes were identified, except for a 2 x 2 m area at the end of the deposit where ten flakes were collected. This area seems to reflect a knapping scatter, as all flakes were of a similar brown chert. Most of the flakes were cortical, and some were broken, so it is difficult to assess the character of the material, though it appears to have Middle Palaeolithic affinities. At site EP19.11 and EP19.12, a very low-density of occasional flakes and cores was identified, with a varying technology and weathering pattern suggesting a broad temporal span for occasional use. At EP19.13, which is located further southwest and into the Ad Dahnā desert, scattered artefacts were identified across a large area, including three Levallois/Levallois-like cores.

## Discussion

Our research provides the first outline of the archaeological record of inland northeast Arabia. This survey complements knowledge from the more intensively studied parts of the peninsula, such as in the Nefud Desert [e.g., 6–8,65–68]. On the one hand, the record of the Al Sulb area shows a recurrent human presence based on archaeological findings from the Lower Palaeolithic to the recent past. However, on the other hand, the remarkably sparse nature of the record is indicative of the limited human use of this landscape over time. For instance, the observation of a single handaxe is in marked contrast to some other parts of Arabia, particularly the west and northwest, where large numbers handaxes are found [e.g., 7,69,37,70]. Likewise, the relatively low reduction intensity is consistent with ephemeral occupations. The resulting simplicity of technology, however, makes chrono-cultural attributions challenging. The sparseness of the record in northeast Arabia presumably reflects the fact that there is, overall, a southwest to northeast cline in humidity in Arabia, with the northeast the furthest from the monsoonal rains which periodically moved inland and ameliorated the region. As is clear with other regions of Arabia [7], the pulses of human occupation identified presumably correlated with occasional windows of increased water availability in the area, aided by the extensive aquifer which makes water accessible even during relatively arid periods. The often highly inverted nature of many of the palaeoriver deposits suggests a very long period of erosion has occurred since their formation, suggesting a great age. However, the presence of numerous channels incised into bedrock suggests that fluvial activity has occurred in the more recent past and these channels are probably ephemerally active during extreme rainfall events such as the one which recently caused erosion and sedimentation within the caves.

The dominant period represented in the regional archaeological record appears to be the Middle Palaeolithic. The most common technology is broadly centripetal flaking, and where retouch is present, it is mostly ‘scraper-like’ in form. These characteristics are most consistent with the MIS 5 period (ca. 130−75 thousand years ago) [7], and distinct from younger MIS 4/3 materials with a focus on unidirectional convergent flaking [71,72]. At this time northeast Arabia would have been on the edge of the monsoonal realm, and the archaeological record suggests limited human use of the landscape. EP19.1 shows that some larger sites are present in the area, associated with prominent raw material outcrops and water sources. Hopefully, future research can apply chronometric dating to Pleistocene archaeological sites in the area. The somewhat generic character of most of the lithics does also mean some caution should be exercised in determining their probable age. We note that coastal Ubaid sites seem to mostly emphasise single platform reduction and contain other elements which we did not identify in the As Sulb area, such as arrowheads and foliates [29,73].

Subsequently, in the Holocene, ephemeral use of the area is shown by findings such as the EP19.90 pendant tomb and the presence of pottery at some sites. This evidence is again faint, with little to connect it to the Ubaid sites known from the coast. This indicates that this arid region separated the coastally orientated Ubaid groups [e.g., 27] from the culturally distinct groups in areas such as northwest Arabia with their unique mustatil structures and other distinctive features [e.g., 20,74,64]. In both northwest Arabia and the Gulf coast, Neolithic activity peaks at a similar time, broadly seven thousand years ago, yet the two spheres seem to have remained distinct. An important aspect of the cultural geography of prehistoric Arabia therefore emerges from our findings, with northeast Arabia perhaps an arid barrier between these different societies.

Our work emphasises the significant potential for Arabian caves to contain faunal assemblages. The caves we investigated contain thousands of exceptionally preserved bones, making them a profoundly important archive of past biodiversity. However, we also show that diverse processes – such as flooding, and recent human activity – influence the character of these assemblages. Radiocarbon dates showed bones on the surface of cave passages range from the recent past to more than three thousand years ago. We are confident that with further dating efforts, and the recovery of buried bones, an even greater chronological span and taxonomic diversity will be revealed, as was found at the Umm Jirsan lava tube to the south where some bones dated to ~7,000 years ago [43]. Given the propensity of many of the caves to flood, efforts should focus on passages not impacted by floodwaters. We found that most of the bones on the surfaces of the cave passages that we surveyed are domesticates, particularly camel. On the other hand, a diverse range of wild species are present in the faunal assemblages. Given ongoing conservation and rewilding efforts, the regionally unique preservation in underground settings presents a crucial source of data. The dominance of domesticates such as camel may mean that humans introduced a significantly greater amount of food for carnivores. However, given the preservation potential of these caves future work may identify rich records of wild fauna from earlier periods. Given the central importance of camels in late Holocene Arabian societies, our findings demonstrate the potential for detailed studies to be conducted on camel domestication and change over time. Further studies in Arabia can continue to elucidate long-term human environment interactions at a crucial geographical nexus between Africa and the rest of Eurasia [e.g., 1,7,26, 75,76].

## Supporting information

S1 Data Appendix: List of macrofauna skeletal remains for Murubbeh Cave, EP19.8, and EP19.91. (XLSX)
